# Insights into Insulin Resistance and Calcification in the Myocardium in Type 2 Diabetes: A Coronary Artery Analysis

**DOI:** 10.3390/ijms24043250

**Published:** 2023-02-07

**Authors:** Queralt Martín-Saladich, Rafael Simó, Santiago Aguadé-Bruix, Olga Simó-Servat, Carolina Aparicio-Gómez, Cristina Hernández, Clara Ramirez-Serra, María Nazarena Pizzi, Albert Roque, Miguel A. González Ballester, José Raul Herance

**Affiliations:** 1 Medical Molecular Imaging Research Group, Nuclear Medicine, Radiology and Cardiology Departments, Vall d’Hebron Research Institute (VHIR), Vall d’Hebron University Hospital, Autonomous University Barcelona, 08035 Barcelona, Spain; 2 BCN Medtech, Department of Information and Communication Technologies, Pompeu Fabra University, 08018 Barcelona, Spain; 3 Diabetes and Metabolism Research Group, VHIR, Department of Endocrinology, Vall d’Hebron University Hospital, Autonomous University Barcelona, 08035 Barcelona, Spain; 4 Centro de Investigación Biomédica en Red de Diabetes y Enfermedades Metabólicas Asociadas (CIBERDEM), Instituto de Salud Carlos III, 28029 Madrid, Spain; 5 Clinical Biochemistry Research Group, Vall d’Hebron Research Institute (VHIR), Biochemical Core Facilities, Vall d’Hebron University Hospital, Autonomous University Barcelona, 08035 Barcelona, Spain; 6 Department of Medicine, Autonomous University of Barcelona, 08193 Barcelona, Spain; 7 Cardiology Department, Vall d’Hebron Barcelona Hospital Campus, Vall d’Hebron University Hospital, 08035 Barcelona, Spain; 8 Radiology Department, Vall d’Hebron Barcelona Hospital Campus, Vall d’Hebron University Hospital, 08035 Barcelona, Spain; 9 Catalan Institution for Research and Advanced Studies (ICREA), 08010 Barcelona, Spain; 10 Centro de Investigación Biomédica en Red de Bioingeniería, Biomateriales y Nanomedicina (CIBERBBN), Instituto de Salud Carlos III, 28029 Madrid, Spain

**Keywords:** myocardium, insulin sensibility, type 2 diabetes, [^18^F]FDG-PET imaging, cardiovascular risk, coronary artery, metabolic disease, computed tomography, calcification

## Abstract

Type 2 diabetes (T2D) is responsible for high incidence of cardiovascular (CV) complications leading to heart failure. Coronary artery region-specific metabolic and structural assessment could provide deeper insight into the extent of the disease and help prevent adverse cardiac events. Therefore, in this study, we aimed at investigating such myocardial dynamics for the first time in insulin-sensitive (mIS) and insulin-resistant (mIR) T2D patients. We targeted global and region-specific variations using insulin sensitivity (IS) and coronary artery calcifications (CACs) as CV risk factor in T2D patients. IS was computed using myocardial segmentation approaches at both baseline and after an hyperglycemic–insulinemic clamp (HEC) on [^18^F]FDG-PET images using the standardized uptake value (SUV) (ΔSUV = SUV_HEC_ − SUV_BASELINE_) and calcifications using CT Calcium Scoring. Results suggest that some communicating pathways between response to insulin and calcification are present in the myocardium, whilst differences between coronary arteries were only observed in the mIS cohort. Risk indicators were mostly observed for mIR and highly calcified subjects, which supports previously stated findings that exhibit a distinguished exposure depending on the impairment of response to insulin, while projecting added potential complications due to arterial obstruction. Moreover, a pattern relating calcification and T2D phenotypes was observed suggesting the avoidance of insulin treatment in mIS but its endorsement in mIR subjects. The right coronary artery displayed more ΔSUV, whilst plaque was more present in the circumflex. However, differences between phenotypes, and therefore CV risk, were associated to left descending artery (LAD) translating into higher CACs regarding IR, which could explain why insulin treatment was effective for LAD at the expense of higher likelihood of plaque accumulation. Personalized approaches to assess T2D may lead to more efficient treatments and risk-prevention strategies.

## 1. Introduction

Type 2 Diabetes (T2D) is a metabolic disorder triggered mainly by sedentary lifestyle and poor dietary habits due to impaired signaling insulin resistance (IR) [1]. One of its main characteristics is high plasma blood glucose levels or hyperglycaemia [2] caused mostly by an insufficient capability of insulin to transport glucose from the blood to the cells, known as IR [3]. Such IR has been postulated as one of the main risk factors for triggering comorbidities in T2D, as the metabolism uses other energetic fuels such as free fatty acids while not being able to appropriately respond to insulin, i.e., impaired insulin sensibility (IS) [4]. In parallel, IR has an effect on the liver as well, enhancing its gluconeogenesis and decreasing cellular glucose uptake in the liver, the muscle and the adipose tissue [5].

Moreover, hyperglycaemia can contribute to overproduction of oxidative features that accelerate inflammation processes and reduce IS in the cells, i.e., decrease cellular glucose uptake for energetic purposes, amongst other problems [6]. Therefore, the molecular link between oxidative stress, IR and T2D has been proposed as a target for new therapies against CVD [7,8,9,10]. In parallel, it has been presented that T2D coexists with coronary artery calcifications (CACs), which has been proposed as a reproducible and widely available approach for assessing CV risk due to plaque accumulation, also known as atherosclerosis [11]. The pathophysiology of T2D namely leads to alterations in the structure and functioning of myocardium that can act as risk factors for CV events, which are the main worldwide causes for prevalence of death in T2D [12,13].

Myocardial glucose metabolism can be assessed using ^18^F-fluoro-deoxyglucose positron emission tomography ([^18^F]FDG-PET), which allows to obtain reliable information on tissular impairment in terms of [^18^F]FDG uptake [14]. In addition, under T2D conditions, the glucose uptake of tissues and organs are diminished due to IR or impaired IS, for which a hyperinsulinemic euglycemic clamp (HEC) is often used. Tissue/organ-specific IS may be quantified through two different PET approaches for myocardial glucose metabolism assessment. The former uses a dynamic PET and performs the approximation of associating IR with a decrease in the myocardial metabolic rate of glucose against healthy controls after performing HEC, where T2D patients showed a reduction in the metabolic rate due to myocardial IR [15,16,17]. The latter and the one used in this study measures the difference in standardized uptake value (ΔSUV = SUV_HEC_ − SUV_BASELINE_) in organs and tissues such as the myocardium by means of the increment of [^18^F]FDG uptake by PET imaging after the HEC procedure compared to the baseline image [18,19,20]. This is a direct quantification of myocardial IS since patients with T2D display residual myocardial glucose uptake in baseline conditions. In addition, the relevance of using this approach is that each patient is their own control, and it is not necessary to use healthy controls, which entails various advantages, among other things.

Thus, previous studies conducted within our research group have unveiled two phenotypes of T2D according to the presence (mIS) or absence (mIR) of myocardial [^18^F]FDG uptake in PET after applying a HEC to T2D patients [18], which relates to the quantified ΔSUV (further developed in the Methods section). Moreover, findings described those mIR patients are linked to higher CACs than mIS subjects in T2D. However, information on calcification and IR relating to each coronary artery territory per each cohort would provide an interesting and more specific approach to the myocardial ΔSUV vs. CACs relationship for clinically relevant purposes. To our knowledge, this has not been yet addressed in the literature due to the absence of proper in vivo methods to measure myocardial IR, for which this topic will also be targeted in the present study.

Unfortunately, current techniques to measure myocardial IR are still far behind from clinical necessities for optimal patient management [21]. In addition, approaches lack information on specific myocardial region affectation and the alterations in the glucose metabolism and structure of the cardiac muscle. In fact, all studies have been performed with systemic IR indexes whilst our group has determined two phenotypes that have not previously been described in the literature, namely the mIS phenotype not associated to the systemic HOMA-IR index [18]. Taking this into account, up until now, the relationship between specific myocardial IS and CACs for different coronary artery regions has not been addressed neither for the general population nor for the T2D phenotypes due to the mentioned absence of methodologies for measuring myocardial IR. In fact, we have recently developed an algorithm to determine myocardial IR based on liver tests, but it has not yet been validated [19]. On the other hand, Fadini et al. proposed two directions regarding insulin dynamics at a cellular level, one relating to glucose uptake and the other to osteogenesis and therefore plaque accumulation [22]. However, previous studies have not addressed the effects on patient tissue or investigated the ways in which this affects T2D subjects, nor have they studied the effect on both T2D phenotypes. Namely, prior published works have not been developed with the aim of determining the role of myocardial IR in the mentioned cohorts.

We suggest that further understanding of the relationships between (a) myocardial IR and coronary artery territories’ CACs, and (b) the effect of insulin in the myocardial tissues regarding (i) different arterial territories and (ii) each T2D phenotype would also provide an insight into personalized management of T2D. Thus, the current pilot clinical trial aimed at untangling the link between ΔSUV and CACs in T2D through a coronary artery region-specific analysis based on myocardial image features on [^18^F]FDG PET/CT. To our knowledge, this study would be the first to target ΔSUV and CACs as independent contributors to CV risk in T2D. Furthermore, this work is the first to tackle the insulin treatment effect on different T2D phenotypes according to its effects on IR and calcification.

## 2. Results

### 2.1. ΔSUV in Myocardial Territories

Results regarding response to insulin in the myocardium have shown different behavior per each of the two phenotypes. Segregation between both proposed phenotypes (mIR and mIS) resulted in a ΔSUV threshold value of 5.01, with AUC = 1.00, *r* = 0.85, and *p* = 2 × 10^−11^. When analyzing each coronary artery region, ΔSUV also displayed observable differences within each phenotype as well. Namely, ΔSUV value was higher in the right coronary artery (RCA), followed by that of the left circumflex (LCX) and eventually the left descending artery (LAD) for both T2D phenotypes (Supplementary Materials, Figure S1). However, differences in ΔSUV values between coronary artery regions within each phenotype displayed distinct behaviors for mIR and mIS regarding the presented parameters, with mIS subjects the only ones showing differences between all regions according to ΔSUV (*p* = 0.016).

### 2.2. CACs in Myocardial Territories

Previous findings showed that mIR patients displayed higher CACs than mIS patients [18]. However, we aimed to evaluate the differences by arterial territories for deeper understanding. Accordingly, we further evaluated the CaScore of each coronary artery territory as the percentage of patients that displayed an accumulated value above three proposed reference values of CV risk: 100, 300 and 400 AU, which have been widely discussed in the literature [23,24,25,26,27]. In fact, we attempted to test the best CACs segregation value between T2D phenotypes. This was achieved by a classification task described in the methods which selected 314 AU as the optimal segregation value between mIR and mIS. Thus, 300 AU was selected as the reference and the other two as the habitual CaScore classification in patients. Differences between mIR and mIS subjects were observed for all coronary artery territories, with mIR patients having the highest calcification incidence when compared to mIS ones.

**Figure 1:**
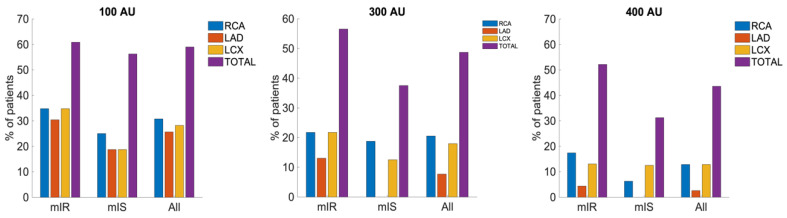
Percentage of patients per group with calcified territories above the reference values of 100, 300, and 400 AU. Group differences between mIR and mIS subjects were not significant for any of the proposed thresholds between regions (*p* > 0.05). Furthermore, when assessing the differences between phenotypes regarding total myocardial CaScore values, *p*-values were determined to be insignificant as well.

When observing the percentage of patients with CACs above 100, 300 or 400 AU, as seen in Figure 1, the same sorting of affected arterial territories was exhibited for all CaScore thresholds in mIR: RCA > LCX > LAD. For 100 and 300, the same was observed for mIS, whereas analysis of 400 showed a different order: LCX > RCA > LAD. Associated to that, when evaluating the average CaScore value inside each artery for the case of both T2D phenotypes, results displayed highest AU in LCX, followed by RCA and LAD (Supplementary Materials, Figure S2). Despite this, when evaluating the changes between mIR and mIS, the order was different: LAD > LCX > RCA (Table 1).

In terms of plaque accumulation, highest incidence was detected in LCX and RCA territories for all cases, although biggest changes between both phenotypes were observed for LAD.

Specifically, for the threshold of 100 AU, RCA seemed to be the most affected coronary artery in mIS, whilst LAD and LCX displayed the same number of patients with CACs above 100 AU. For mIR, RCA and LCX showed a higher number of patients with CACs > 100 AU compared to LAD. Nonetheless, when increasing the cut-off value to 300 AU, a change in distribution was not observed, although LAD did not show with CACs > 300 AU for the case of mIS. In contrast, when analyzing the changes associated to increasing the cut-off value further to 400 AU, a prominent reduction in the affected patients at the mIS cohort was observed, but accumulation was still present in the mIR group with RCA being the most affected artery, followed by LCX and LAD, respectively.

### 2.3. ΔSUV vs. CACs in Myocardial Territories

Comparison of each artery territory in terms of ΔSUV and calcifications was assessed, which exhibited a trend of decreasing IS when comparing mIS to mIR, RCA displaying the most prominent change as it has been mentioned above, followed by LCX and LAD, respectively. The evolution of CACs per each group and artery was assessed according to the number of patients that displayed calcifications above or below 300 AU, respectively. A different tendency was observed regarding plaque accumulation, with LAD showing the biggest increase, followed by LCX and RCA, as seen in Table 1. In terms of ΔSUV, smallest changes from mIS to mIR were shown for LAD, whilst LAD showed the greatest increase in calcification between phenotypes.

In parallel, the changes in [^18^F]FDG uptake relating to plaque accumulation were evaluated. Biggest ratio relating CACs and ΔSUV was observed for LCX in mIR patients whereas RCA had the biggest value for mIS subjects instead. Likewise, the differences between mIS and mIR in the CACs/ΔSUV ratio displayed that increased CACs were linked to increased IR (decreased ΔSUV), and more specifically associated to the left side of the heart which displayed bigger changes (LAD and LCX). In fact, LAD seems to be the territory more affected between mIS and mIR T2D patients, with an increase in CACs and a decrease in ΔSUV.

### 2.4. Effect of Insulin Treatment in Both T2D Myocardial Phenotypes

Following the differentiated T2D phenotypes (mIR or mIS), a different pattern regarding the incidence of IR and plaque accumulation based on insulin treatment was observed as seen in Figure S3 (Supplementary Materials) and Table 2, respectively. Treatment for each case has also been discussed (Section 5). In terms of ΔSUV, mIR displayed an increase in [^18^F]FDG uptake for all coronary artery territories when being treated with insulin. However, the effect on mIS was different: insulin treatment resulted in a very low increase in [^18^F]FDG uptake in RCA and a slightly bigger increase on the left side (LAD, LCX).

**Figure 2:**
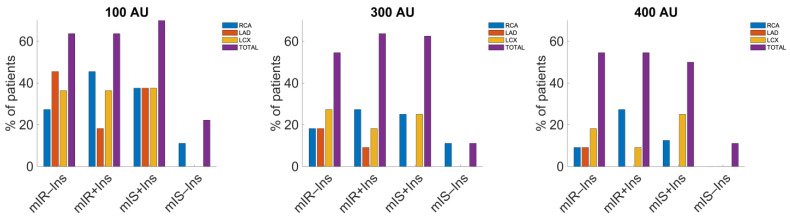
Percentage of patients per phenotype with/without insulin treatment group with affected regions above 100, 300, and 400 AU. In the figure, with-insulin (+) and without-insulin (−) treatment is expressed with symbols. Differences between mIR + insulin and mIR − insulin subjects were not significant for any arteries, and neither were they for mIS + insulin and mIS − insulin (*p* > 0.05), although for 300 AU, *p*-values were the lowest. For the case of the established AU reference values and all phenotype ± insulin categories, results did not display statistical significance for 100 AU and 300 AU, *p* > 0.05, but they did for 400 AU, *p* = 0.04.

Analysis of treatment with insulin in each T2D phenotype, i.e., mIR (with/without insulin) and mIS (with/without insulin), displayed different tendencies regarding presence of CACs per each territory (Figure 2). Results showed higher incidence of plaque accumulation in the RCA for mIR with insulin in all three AU cut-off cases. In parallel, LAD showed greater affectation in calcifications for the case of mIR without insulin. Moreover, for mIS, similar tendencies were observed for 100 and 300 AU, with RCA = LCX > LAD being the group treated with insulin more prone to CACs. When comparing mIS with insulin to mIS without insulin, LCX and RCA displayed increased plaque presence. No patients were determined to have CACs above the 400 AU threshold when not treated with insulin in the LAD, whilst an increase in RCA and LCX was observed when insulin intake was present.

Results displaying the effect of insulin treatment on calcification exhibited different behaviors per each myocardial phenotype of T2D patients. For the case of percentage of patients with CACs above 100 AU, results between each phenotype without vs. with insulin treatment showed an increase in the presence of plaque for mIS patients for all regions, and a decrease for mIR in the LAD whilst an increase in RCA. However, when using the 300 AU cut-off, the portion of patients with calcifications in the LCX and LAD above the threshold decreased with the use of insulin in mIR subjects with an increase in RCA, whilst for mIS with vs. without insulin treatment, no changes were displayed in the LAD and an increase in LCX and RCA was observed, as seen in Table 3. Furthermore, the analysis using 400 AU as cut-off exhibited a similar behavior instead, with increased calcifications in the RCA and decreased calcifications for LCX in mIR subjects with insulin treatment, and no changes for LAD but an increase in RCA and LCX in the case of mIS patients with the same treatment.

In terms of accumulated CACs (Supplementary Materials, Figure S4), a decreased amount of plaque was detected for mIR subjects with insulin treatment on the left coronary arteries with an increase in RCA, whereas higher values were seen for all territories in mIS subjects with insulin intake.

Despite insulin intake reaching an increase in [^18^F]FDG uptake in all territories of both phenotypes, changes in [^18^F]FDG uptake were distributed unevenly for both groups of phenotype with/without insulin. In mIR, biggest difference was observed for RCA, followed by LAD and LCX, whereas in mIS, the most prominent change was seen for LAD, followed by LCX and RCA. The biggest changes regarding CACs presence for mIR were observed for increasing RCA, followed by decreasing LCX and LAD according to the 300 AU cut-off. For mIS, the order was different, with LCX having more patients CACs > 300 AU followed by RCA and LAD. Thus, insulin treatment resulted in an increase in CACs regarding IS in RCA in both phenotypes, with a more significant effect on mIS than on mIR. At the same time, a more positive effect on IS in RCA was observed for mIR rather than mIS instead.

## 3. Discussion

### 3.1. ΔSUV in Myocardial Territories

In terms of response to insulin, RCA had the highest ΔSUV values whilst LAD showed the lowest values for both T2D phenotypes. LAD is responsible for approximately half of the portion of blood supply to the LV. Thus, this finding has shown that in terms of myocardial IR, a critical endangerment and CV risk could be associated to impaired IS in such coronary artery territory in T2D. In fact, alterations in LAD propitiated a non-favorable outcome [28]. When the coronary artery territories of both phenotypes were compared, RCA displayed the biggest decrease in IS from mIS to mIR, which translates into higher IR contribution in this territory. Thus, it is the most affected by glucose-related metabolic disruptions within the studied patients in spite of showing the biggest [^18^F]FDG uptake values in the general T2D population assessment.

### 3.2. CACs in Myocardial Territories

Findings within the study propose using 314 AU as the optimal reference value to properly stratify T2D patients according to myocardial IR in terms of mIR and mIS. This value arises from a classification task described in the methods. Thus, of all the presented cut-off values, 300 AU was determined to be the optimal reference value between phenotypes, which supports previous statements proposing the capability of using such as an alternative for CV risk assessment [26]. Moreover, it has a higher potential than thresholds of 100 and 400 for the myocardium proposed by other authors [23,24,25,26,27]. However, analysis using all thresholds has been conducted in the current study, showing similar results for all of them.

When interpreting the percentage of patients with an affected coronary artery territory above each reference value (100, 300 and 400 AU) [23,24,25,26,27], a differentiated distribution for each phenotype was observed. mIR patients showed a higher number of subjects with CACs above each AU cut-off. Likewise, findings propose that higher exposure to CV complications due to arterial obstruction in terms of calcification is to be expected for mIR individuals, the RCA being the biggest contributor to IR and also the artery where more calcifications are usually detected in patients. However, in terms of calcifications linked to IR, RCA has also been determined to be the least changing territory when changing from mIR to mIS, making it exposed to lower risk regarding IR. Despite LAD being the most common location for obstruction in the general and T2D population [29], in the present findings, a somewhat opposite behavior has been observed for T2D subjects which showed this territory as the least affected in general terms. However, when contemplating the differentiation between mIR and mIS, LAD was in fact the artery that showed the biggest differences between both phenotypes in terms of CACs. Therefore, the change from the mIS to the mIR phenotype causes more changes in LAD, the artery that is usually obstructed in patients, for which the differences between myocardial phenotypes in T2D are associated with CV risk. Therefore, we propose that LAD should be targeted for obstruction prevention in T2D population specifically for mIR cases.

### 3.3. ΔSUV vs. CACs in Myocardial Territories

Analysis linking ΔSUV and CACs displayed different evolutions for each arterial region when comparing mIR and mIS phenotypes. Higher myocardial IR when switching from mIS to mIR was observed for RCA, whilst LAD showed the greatest increase in calcification between phenotypes instead. However, after analyzing the CACs/ΔSUV ratio, LAD appears to be the territory more affected in terms of calcification related to myocardial IR between both groups of patients. Considering this, it could explain why LAD is determined to be the most affecting territory by occlusion linked to IR in T2D patients whilst being the least affected by IR. Since it is the territory with biggest changes between mIR (high-risk) and mIS (low-risk) subjects, we propose LAD as the leading artery in terms of risk exposure in T2D due to myocardial IR. In addition, this displays the relevance of differentiating between mIR and mIS for optimal patient management regarding CV risk factors as myocardial IR and plaque accumulation.

### 3.4. Effect of Insulin Treatment in Both T2D Myocardial Phenotypes

According to the obtained data relating to calcification as CV risk exposure and ΔSUV-related phenotypes, a pattern displaying a different tendency for plaque accumulation in the coronary arteries for each phenotype was observed in terms of insulin treatment. Differences between treatment groups (phenotype with/without insulin) in terms of ΔSUV were not as prominent as in the CACs analysis. ΔSUV increased substantially for the patients treated with insulin in the mIR for all coronary artery regions, whereas the changes in mIS were minimal. Insulin treatment for mIR patients resulted in increased CACs at RCA and decreased for LCX and LAD, whilst an increase in RCA and LCX was observed for mIS subjects instead.

Curiously, mIR subjects without such treatment were more likely to develop calcification when compared to those who were treated with insulin, whereas mIR patients with insulin displayed an increased ΔSUV. Likewise, insulin treatment for mIR subjects decreased the risk for arterial obstruction whilst providing superior sensitivity to insulin. In parallel, mIS patients followed an opposite trend: as the ones treated with insulin, they were more prone to plaque accumulation rather than those who were not taking it, whilst IS was not as concentrated under insulin treatment conditions. This interesting and novel finding per each phenotype suggests that treatment should be approached differently depending on whether the patient under evaluation belongs to the mIR or mIS T2D subgroup to avoid CACs. Furthermore, if treatment were to be applied indistinct of phenotype, some patients could improve IS by responding to the insulin intake (mIR) at the expense of other patients being exposed to a higher likelihood for suffering from calcifications (mIS). Following such statements, we believe there is a need for further research regarding biochemical alterations relating to ΔSUV and calcification, specifically for each phenotype of T2D. Nonetheless, other publications have addressed the effects of anti-diabetic drugs in calcification but only at a cellular level [30,31,32], for which we propose that further research at a tissular or organ level should be performed as well.

In parallel, it has been observed that insulin supplements influence glucose uptake and plaque accumulation, which is in consonance with previously published studies proposing a duality in insulin function regarding either tissular glucose uptake (good) or calcification (bad) [22]. For the former, compensation of high blood glucose levels via insulin secretion eventually results in the binding of glucose transporters into the cell due to the insulin receptor substrates, which therefore enhance glucose uptake. However, the latter mechanism can be stimulated by the presence of such substrates promoting the differentiation of smooth muscle cells into osteogenic cells contributing to atherogenesis, among many other factors [30,33,34]. Furthermore, it has been proposed that there exist exceptions regarding the presented pattern. For instance, mIR patients without insulin treatment that had an intake of vitamin D or calcium usage helpers did not develop calcifications (two individuals), whilst two mIR subjects without insulin input but with calcium supplements did show accumulated plaque. It has been proposed that vitamin D targets vascular tissue, as the output hormone from its metabolization has been determined to prevent plaque formation due to inhibition bone morphogenic proteins and inflammatory cytokines as well as being linked to up-regulation of calcification inhibitors [35]. Moreover, it has also been proposed that it helps with plasma calcium absorption to bones [36]. Likewise, presented results are backed by the previously published works, for which assumptions are being made. Due to this, further studies are mandatory to elucidate the observations of the current pilot clinical trial.

### 3.5. Limitations of the Present Study

We acknowledge that the present study has several limitations that should be addressed in the near future regarding the interactions of different treatment drugs with insulin-related plaque accumulation. As proposed by previous works [37,38,39], antidiabetic and antihypertensive drugs could have interfered with the myocardial glucose metabolism and therefore their relationship with T2D-related CACs should be independently studied. Moreover, antiaggregant therapy could be useful to avoid CACs and presents itself as a very good alternative to avoid the counter-effects of insulin intake [40]. However, the drugs displayed in table (in Section 4.5) did not show statistical significance according to Fisher’s test between mIR/mIS and with/without treatment *p* > 0.05. Thus, we believe that the presented results are of a reliable nature and not significantly altered by antidiabetic and anti-hypertensive treatments. Nonetheless, we suggest further trials and analyses should be performed to clarify the potentiality of such approach.

## 4. Materials and Methods

### 4.1. Patient Characteristics

The images used in this study were acquired through a clinical trial which comprised 42 T2D subjects while being conducted according to the tenets of the Helsinki Declaration. The Ethic Committee of the Vall d’Hebrón University Hospital approved all procedures (protocol number PR(AG)01/2017).

The inclusion criteria for the patient set were: (1) diagnosis of T2DM from at least 5 years before the screening, (2) patients with T2DM controlled from at least a year before the scanning, (3) age from 50 to 79 years old, (4) normal ECGs. The exclusion criteria were: (1) type 1 diabetes, (2) any previous CV event, (3) any contraindication or claustrophobia for the PET/CT, (4) any concomitant pathology related to a short life expectancy, (5) daily alcohol drinkers, and (6) smokers who did not stop smoking at least ≤1 year prior to recruitment.

For each patient, two [^18^F]FDG-PET images were acquired before and after the HEC, the latter protocol performed as described by our group [18,19] and Gerber et al. [14,41]. Briefly, patients were laid on a stretcher and two catheters were inserted in the antecubital vein of each arm, the left for arterialized venous blood sampling and the right for insulin and glucose infusion, where [^18^F]FDG was also administered. The corporal surface of each patient was obtained after applying the following equation:

$$\mathrm{Corporal}\;\mathrm{surface}=\sqrt{\frac{\left(\mathrm{weight}\left(\mathrm{kg}\right)\times \mathrm{height}\left(\mathrm{cm}\right)\right)}{3600}}.$$

The infusion rate of 1 UI/mL insulin in physiological solution per patient was determined using the following formula:

$$\mathrm{mUI}/\left(m^{2}\times \min\;\right)\times \left(1\;\mathrm{UI}\right)/\left(1000\;\mathrm{mUI}\;\right)\times \left(60\;\min\right)/\left(1\;h\;\right)\times \mathrm{Corporal}\;\mathrm{surface}\;\left(m^{2}\right)=\mathrm{UI}/h.$$

The infusion rate of 10% glucose in physiological solution was calculated using the following formula:

$$\left(2\;\mathrm{mg}\right)/\left(\mathrm{kg}\times \min\;\right)\times \left(1\;\mathrm{mL}\right)/\left(100\;\mathrm{mg}\;\right)\times \left(60\;\min\right)/\left(1\;h\;\right)\times \mathrm{Weight}\;\left(\mathrm{kg}\right)=\mathrm{mL}/h.$$

After 5 min of insulin infusion, its rate was reduced to approximately 25% and the glucose infusion was initiated. After ten minutes, glucose infusion rate was adjusted taking into account the plasma concentration of glucose, measured each five minutes by using a glucometer Accu-check Aviva. At the same time, the insulin infusion rate was reduced by approximately 50% from the initial rate and was maintained constantly during the rest of the HEC procedure. When plasma glucose concentration was stabilized after three constant consecutive measurements which did not differ from each other by more than 10% and at least after 1.5 h of applying HEC procedure in patients, [^18^F]FDG was intravenously administered. Then, HEC was maintained during the entire [^18^F]FDG uptake period. Sixty minutes later, clamping was aborted, and the patient underwent PET evaluation.

Whole-body IS was assessed by measuring the mean glucose infusion rate during the last 40 min, as proposed by Moreno-Navarrete et al. [42].

Moreover, baseline blood samples were analysed at the Biochemistry Core Facilities of Vall d’Hebron University Hospital using standardized and validated routine methodologies to obtain biochemical data as displayed in Table 4. Those were acquired during the baseline stage, i.e., before the HEC, as they corresponded to the true metabolic characterization of the patient.

### 4.2. Imaging

Two [^18^F]FDG-PET/CT studies per patient were performed in a random order within 2 days with at least 8 h of fasting condition and 24 h after stopping medication intake. A total of 1.9 MBq/Kg of [^18^F]FDG was administered to each patient at each scanning session and imaging was performed at 6 min of cardiac bed. In the HEC imaging, [^18^F]FDG was administered after at least 1.5 h from the beginning of the protocol. Image acquisition was performed in a Biograph mCT 64S scanner and image reconstruction was performed by means of Gaussian filtering (order 3) with 3 iterations and 21 subsets. ZOOM value 2 was applied, with pixel spacing measuring 1.59095 × 1.59095 mm and matrix size of 256^2^ with a slice thickness of 2.027 mm.

After the PET image was acquired, coronary synchronized CT calcium score data were obtained with specifications of tube voltage = 80 kV, pitch = 0.9 pixel, spacing = 0.7168 mm isotropic, tube current = 126 mA, exposure time = 0.5 s, image matrix size = 512^2^ and slice thickness = 0.6 mm.

### 4.3. Myocardial Segmentation

The most extended model for the myocardium is the AHA-17 segment model for the heart [43], which comprises 17 segments distributed in 2 fractions of 35% for the basal and mid-cavity, respectively, and 1 fraction with the remaining 30% for the apical section. Moreover, a three-way differentiation regarding the coronary artery blood supply is also followed depending on the proximity of each segment to the closest coronary artery (including right (RCA), left descending (LAD) and left circumflex (RCA)) using the standard AHA recommendations for polar map segments to the three vascular territories.

Semi-automatic image segmentation and processing was performed using the AHA-17 [44] polar map segments of the PCARD configuration from the PMOD software [45] (used in this study) that provide quantitative data of each segment within the organ, which is why they are extendedly used within the research community [46,47]. To calculate the myocardial glucose metabolism, we segmented each territory on both pre- and post-HEC images and obtained the glucose uptake data in terms of SUV. We then subtracted SUV_HEC_ − SUV_baseline_ to obtain the ∆SUV (or IS) of the corresponding region.

For the case of CACs, calcium scores were determined using a semi-automatic segmentation methodology by means of the syngo.via cardiac CT software according to each coronary artery as well.

### 4.4. Phenotype Definition

Two phenotypes in terms of myocardial IR, mIR and mIS, have been previously described by our group according to the T2D population [18,19], with mIR showing higher CV risk exposure due to IR and plaque accumulation. The definition of mIR and mIS was addressed by determining the ΔSUV by comparing the [^18^F]FDG-PET images before and after the HEC protocol. The selection of the patients was carried out by specialists in nuclear cardiology (M.N.P, A.R. and S.A-B.) according to whether they visually observed an increase in myocardial glucose uptake after HEC or not. Thus, 16 patients displayed enhanced myocardial activity after the HEC, whereas the remaining 26 showed non-observable glucose uptake instead. This separation exhibited the functional differences between both groups, which resulted in the definition of mIS and mIR phenotypes, respectively. In terms of whole-body IS, the values per group were of 1.46 ± 0.77 for mIR and 1.81 ± 0.86 for mIS (*p* = 0.03).

### 4.5. Medication

Patients included in the trial were receiving different treatment drugs as seen in Table 5, mostly for diabetes and high blood pressure. Those included metformin/pioglitazone (control blood sugar levels), insulin (control blood sugar levels by promoting glucose uptake in the cell), statins (lower the level of low-density lipoprotein cholesterol in the blood), β-blockers (treat high blood pressure), DDP4-inhibitor (control blood sugar levels), vitamin D/calcium (VitD/Ca^2+^) usage helper (regulate calcium absorption in the body), calcium supplements (treat osteoporosis), Ca^2+^ channel blockers (treat high blood pressure), and GLP-1 agonists (control blood sugar levels) [48]. The median ± standard deviation (SD) duration of medication with insulin was 10 ± 1.7 years.

### 4.6. Classification Task

We aimed to determine the best differentiation between mIR and mIS in terms of total CACs, i.e., the accumulated plaque in the whole myocardium expressed in Agatston units (AU). To achieve this, receiver operating curves (ROC) and the corresponding area under the curve (AUC) values were obtained for the total CACs of the patient by using the presence (1) or absence (0) of uptake in the HEC as the response variable corresponding to mIS and mIR cohorts, respectively. The optimal cut-off was determined to be 314 AU.

### 4.7. Statistical Analysis

For statistical analysis and plotting, MATLAB R2020b [49] was used. Different surveys were performed in the presented work, including the analysis of ΔSUV at a global and a regional level, as well as the interrelationships between ΔSUV and CACs. Bar plots with standard errors were displayed for comparison purposes and to assess differences between arterial territories. The same analysis was performed for comparison of T2D phenotypes and insulin treatment. For the appraisal of the association between both image-based features (ΔSUV and CACs), Spearman correlation coefficients (r) were acquired for the link between each of the studied variables. *p*-values were obtained for each statistical test, considered significant if p<0.05 with a confidence interval of 95%. Differences between groups were computed by means of Mann–Whitney (two-sample) or Kruskal–Wallis (three- or more-sample) analysis for numerical data, and via Fisher’s and Chi-square test for categorical variables using contingency tables ruled by classes relating to clamp uptake presence and plaque accumulation regarding different reference values (100, 300, and 400 AU [23,24,25,26,27]), both for phenotype and treatment comparison.

## 5. Conclusions

With this proof-of-concept study, we provide an insight into personalized treatment that targets specific necessities according to mIR or mIS cohorts. Moreover, differences between phenotypes displayed LAD to be the leading territory in terms of associated CV risk by showing an increase in CACs when changing from low-risk (mIS) to high-risk (mIR) populations. This would confirm that myocardial IR is a CV risk factor caused by CACs. We propose that further studies should be performed to clarify whether treating patients with insulin should be contemplated depending on the T2D phenotype whilst aiming at untangling the mechanisms behind the role of insulin in the ΔSUV–CACs dynamics.

## Figures and Tables

**Table 1:** Comparison of region contributions according to the type of subject. Data exhibited by means of ΔSUV and CACs (percentage of subjects using 300 AU as reference).

	mIR			mIS			mIS-mIR			*p*-Values		
	RCA	LAD	LCX	RCA	LAD	LCX	RCA	LAD	LCX	RCA	LAD	LCX
ΔSUV	0.82	0.38	0.52	9.33	7.49	8.60	8.51	7.11	8.08	8 × 10^−8^	1.4 × 10^−7^	1.0 × 10^−7^
CACs	21.74	13.04	21.74	18.75	0	12.5	−2.99	−13.04	−9.24	0.36	0.11	0.28
CACs/ΔSUV	26.51	34.32	41.81	2.00	0	1.45	−0.35	−1.83	−1.14	-	-	-

**Table 2:** Classification of patients regarding plaque presence. Percentage of patients with any kind of calcification (CACs > 0) according to the phenotype and insulin intake prescription.

	mIR	mIS
Insulin intake	62% have calcifications	88% have calcifications
No insulin intake	85% have calcifications	25% have calcifications

**Table 3:** Comparison of region contributions. Results according to the T2D phenotype and insulin intake prescription by mean of ΔSUV and CACs (percentage of subjects using 300 AU as reference).

	**mIR without Insulin**			**mIR with Insulin**			**mIR with/without**			***p*** **-Values**		
	RCA	LAD	LCX	RCA	LAD	LCX	RCA	LAD	LCX	RCA	LAD	LCX
ΔSUV	0.33	0	0.22	1.21	0.72	0.75	0.88	0.72	0.53	0.76	1.00	0.60
CACs	18.18	18.18	27.27	27.27	9.09	18.18	9.09	−9.09	−9.09	0.69	0.72	0.77
CACs/ΔSUV	55.09	-	123.95	22.54	12.63	24.24	10.33	−12.63	−17.15	-	-	-
	**mIS without Insulin**			**mIS with Insulin**			**mIS with/without**			***p*** **-Values**		
	RCA	LAD	LCX	RCA	LAD	LCX	RCA	LAD	LCX	RCA	LAD	LCX
ΔSUV	8.86	6.68	7.81	8.89	7.57	8.57	0.03	0.89	0.76	0.61	0.48	0.88
CACs	11.11	0	0	25.00	0	25.00	13.89	0	25	0.52	-	0.13
CACs/ΔSUV	1.25	0	0	2.81	0	2.92	463	0	32.89	-	-	-

**Table 4:** Patient characteristics. Data indicated as median ± interquartile range (IQR), except for age that has been shown as median ± SD.

Parameter	Median		IQR
Age	66	±	7
HbA1c (%)	7.30	±	0.95
Creatine (mg/dL)	0.74	±	0.31
Bilirubin (mg/dL)	0.54	±	0.29
Sodium (mmol/L)	138.25	±	3.68
Potassium (mmol/L)	4.23	±	0.47
Calcium (mmol/L)	9.6	±	0.5
Aspartate aminotransferase (UI/L)	23	±	14
Alanine aminotransferase (UI/L)	21	±	18
Alkaline phosphatase (UI/L)	73	±	34
Gamma glutamyl transferase (UI/L)	27	±	22
Peptide C (ng/mL)	1.81	±	1.80
Leptin	30.50	±	34.93
Interleukin-6 (pg/mL)	2.76	±	3.04
SFRP-1 (ng/mL)	0.71	±	0.52
Whole-body IS	1.55	±	0.72

**Table 5:** Patient medication. Data indicated as total number of subjects (percentage compared to the total of each cohort) and Fisher’s test *p*-values. Although calcium supplements displayed significant differences between both cohorts, we did not exclude the patients from the analysis since the study of medication was performed within each phenotype (mIS and mIR) and not for the whole T2D population.

Medication	All (*n* = 42)	mIS (*n* = 16)	mIR (*n* = 26)	*p*-Value
Metformin/Pioglitazone	27 (64%)	10 (63%)	17 (65%)	1.00
Insulin	21 (50%)	8 (50%)	13 (50%)	1.00
Statins	27 (64%)	10 (63%)	17 (65%)	1.00
β-blockers	29 (69%)	13 (81%)	16 (62%)	0.30
DDP4-inhibitors	19 (45%)	5 (31%)	14 (54%)	0.21
VitD/Ca^2+^ usage helpers	7 (17%)	1 (6%)	6 (23%)	0.22
Calcium supplements	3 (7%)	3 (19%)	0 (0%)	0.049
Ca^2+^ channel blockers	7 (17%)	3 (19%)	4 (15%)	1.00
GLP-1agonists	10 (24%)	3 (19%)	7 (27%)	0.72

## Data Availability

Not applicable.

## Supplementary Materials

The following supporting information can be downloaded at: https://www.mdpi.com/article/10.3390/ijms24043250/s1.
