# Supplementary material for: Insights into Insulin Resistance and Calcification in the Myocardium in Type 2 Diabetes: A Coronary Artery Analysis
Source: Int J Mol Sci. 2023 Feb 7;24(4):3250. doi: 10.3390/ijms24043250 (PMC9959651; doi:10.3390/ijms24043250)
Supplement: Supplementary file 1 [file ijms-24-03250-s001.zip › ijms-2149761-supplementary.pdf]

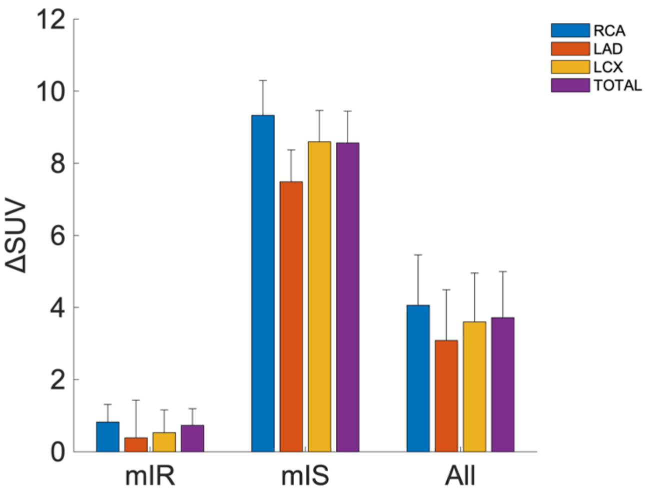

Figure S1. Differences between groups and coronary artery regions according to IS. P-values relating to differences between mIR vs mIS were of 0.005, 0.012, and 0.011 for RCA, LAD, and LCX, respectively. No statistically significant differences were observed between all three territories within phenotypes.

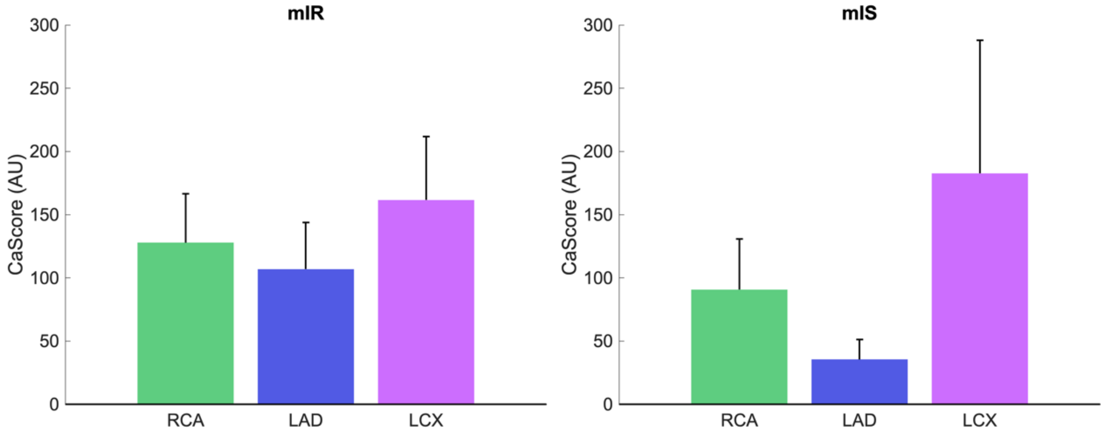

Figure S2. Differences between coronary artery regions in terms of calcification CaScore values in AU. Differences between all three territories within groups were p=0.10 for mIR and p=0.07 for mIS, whilst p-values comparing RCA, LAD, and LCX between phenotypes were p>0.10 instead.

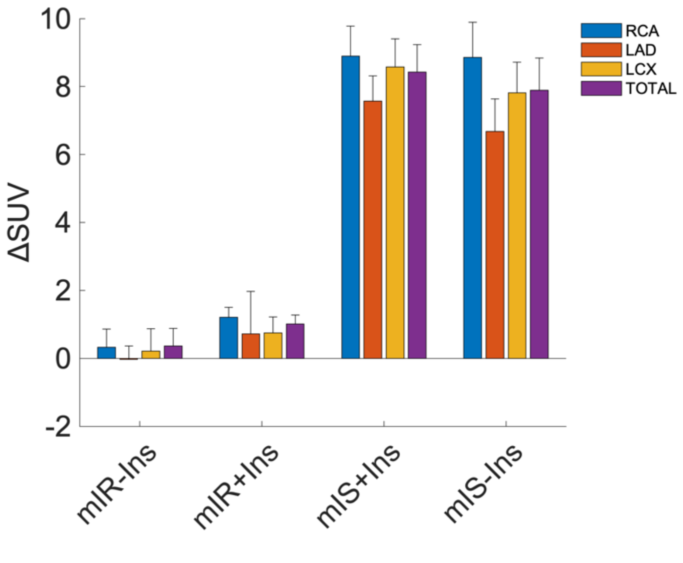

Figure S3. IS for each coronary territory regarding different treatment groups within a phenotype. Differences were not significant (p>0.05) for any territories when comparing a) mIR with/without insulin and b) mIS with/without insulin. Nor they were when comparing territories within a group.

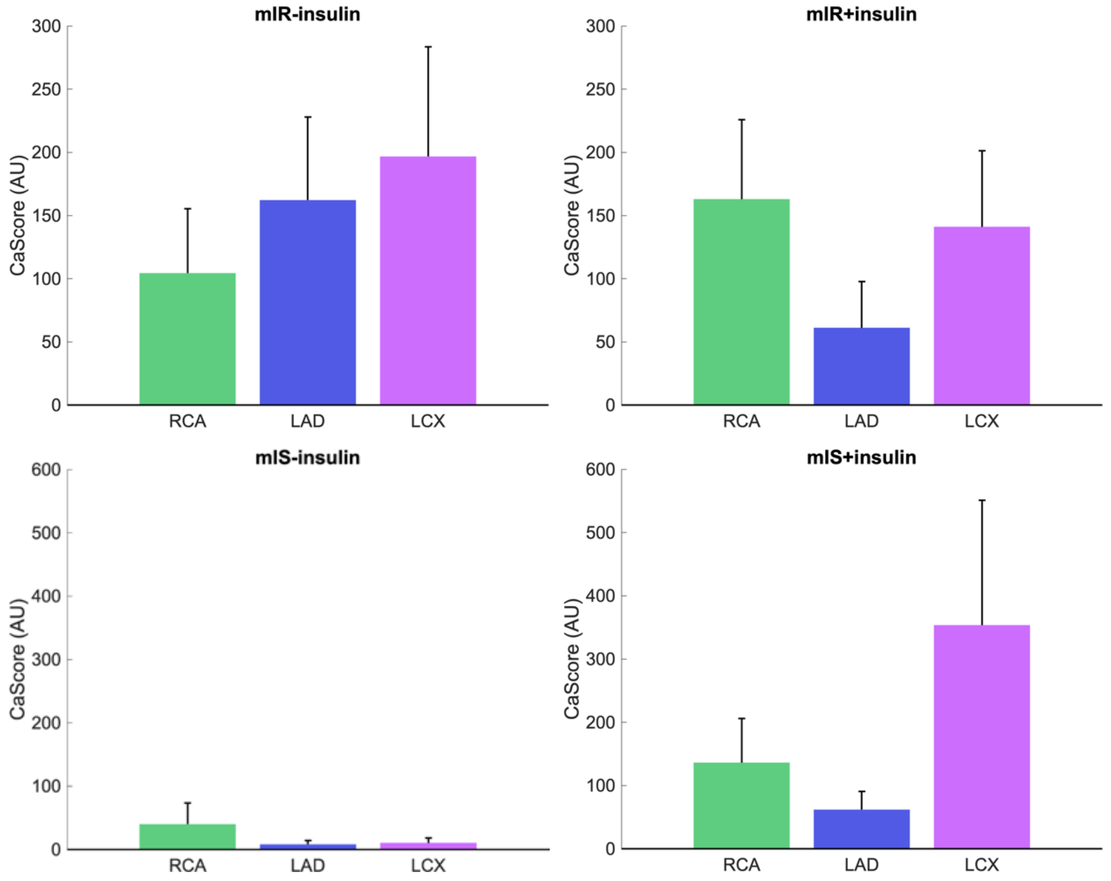

Figure S4. Differences between coronary artery regions in terms of calcification CaScore values in AU. No statistically significant results when comparing territories within a group were found for a) mIR-Insulin, b) mIR+Insulin, c) mIS-Insulin, and d) mIS+Insulin. Differences between RCA, LAD, and LCX for mIS with/without insulin resulted in p=0.06, 0.56, and 0.18; whilst for mIR with/without insulin were of p=0.12, 0.005, and 0.30 instead.
